# Liver X Receptors and Their Implications in the Physiology and Pathology of the Peripheral Nervous System

**DOI:** 10.3390/ijms20174192

**Published:** 2019-08-27

**Authors:** Venkat Krishnan Sundaram, Charbel Massaad, Julien Grenier

**Affiliations:** Faculty of Basic and Biomedical Sciences, Paris Descartes University, INSERM UMRS 1124, 75006 Paris, France

**Keywords:** LXR, oxysterol, cholesterol, peripheral nervous system, myelin, schwann cell

## Abstract

Recent research in the last decade has sought to explore the role and therapeutic potential of Liver X Receptors (LXRs) in the physiology and pathologies of the Peripheral Nervous System. LXRs have been shown to be important in maintaining the redox homeostasis in peripheral nerves for proper myelination, and they regulate ER stress in sensory neurons. Furthermore, LXR stimulation has a positive impact on abrogating the effects of diabetic peripheral neuropathy and obesity-induced allodynia in the Peripheral Nervous System (PNS). This review details these findings and addresses certain important questions that are yet to be answered. The potential roles of LXRs in different cells of the PNS are speculated based on existing knowledge. The review also aims to provide important perspectives for further research in elucidating the role of LXRs and assessing the potential of LXR based therapies to combat pathologies of the Peripheral Nervous System.

## 1. Introduction

Liver X Receptors (LXRs) are ligand-activated transcription factors that exist in 2 isoforms: LXRα and LXRβ encoded in mice by the genes *Nr1h3* and *Nr1h2,* respectively. Although classified as orphan receptors upon discovery, oxidized cholesterol derivatives (oxysterols) such as 20(S)-, 22(R)-, 24(S)-, 25- and 27-hydroxy cholesterol (HC) and 24(S), 25-epoxycholesterols were later found to be their natural ligands [1,2,3,4,5]. Further research in endocrinology over the past two decades has resulted in the identification of many natural and synthetic agonists and antagonists of LXRs with claimed specificities to each of the two isoforms [5].

LXRs have been implicated in several physiological processes such as lipid metabolism and homeostasis, inflammation and cholesterol homeostasis, as well as a plethora of diseases such as Multiple sclerosis, Alzheimer’s, Arthritis, and cancers [6,7,8,9,10]. Similarly, the implications of oxysterols are also equally diversified both in physiological and pathological contexts [11].

The implications of LXRs in both the physiology and pathology of the Central Nervous System (CNS) have been extensively studied and reported over the last few years. Seminal and extensive reviews have diligently detailed almost every aspect of LXRs’ function in the brain from development to diseases [12,13,14]. The implication of LXRs in the Peripheral Nervous System (PNS) is a relatively nascent theme of research that is garnering much attention only in the past decade. This review, therefore, aims to assimilate the existing knowledge and also provide certain perspectives on further exploring the role of LXRs in the PNS.

**Figure 1:**
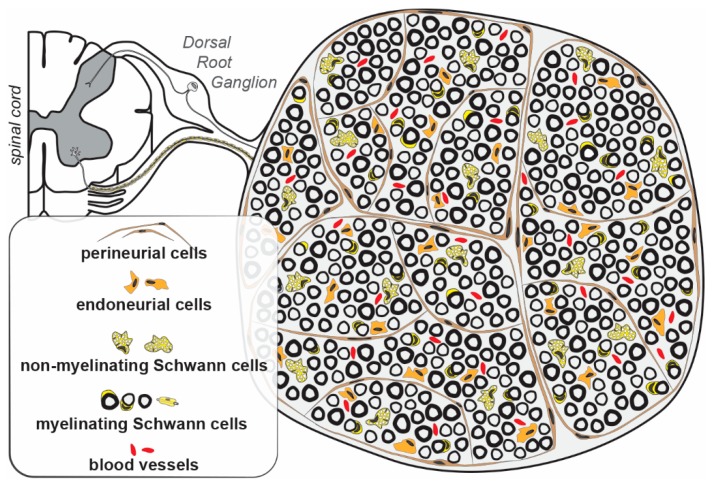
Peripheral nerve cellular structure. Sensory neurons emanating from the dorsal part of the spinal cord with their somata in dorsal root ganglia project their axons towards the spinal cord on the one hand and their peripheral endpoints on the other. Some of these sensory axons are myelinated by Schwann cells, and others are ensheathed by non-myelinating Schwann cells in specific structures called “Remak bundles”. Motor axons emanating from the ventral roots of the spinal cord are wrapped by myelinating Schwann cells. Perineurial cells surround multiple axons and Schwann cells to delineate fascicles. They provide mechanical and structural integrity to these fascicles. Endoneurial cells are dispersed inside the fascicles. They provide major components of the extracellular matrix, perform immune-surveillance, and are implicated in remyelination. The vasculature is composed of endothelial cells surrounded by pericytes that provide the nerve-blood barrier.

A holistic understanding—of the implications of LXRs and their natural ligands—elicits a clear division of the different components of the PNS. Each specific cell type of the PNS functions differently at a molecular level; making the fatty acids, and cholesterol metabolism in the spinal nerves can require highly diverse and cell-type-specific actions. Broadly, the PNS can be classified into the following cellular subtypes: Schwann cells, endoneurial cells, perineurial cells, sensory neurons (and their axons originating at the spinal ganglia) [15] (Figure 1).

The somata of the motor neurons are located in the spinal cord, and only their axons form a part of the PNS. Hence, lipid and cholesterol metabolism, and possible implications of LXRs in motor neuron physiology and associated diseases falls beyond the ambit of this review but have been reviewed elsewhere [16,17,18]. In the following sections, a survey of literature pertaining to different PNS cell types is detailed along with interesting, yet unanswered, questions, and possible future avenues of research.

## 2. Schwann Cells

Schwann cells constitute a major portion of the spinal nerves and are by far the most extensively studied glial cells of the peripheral nervous system. They are directly responsible for the production of the myelin sheath around peripheral axons [19]. The composition of myelin and the underlying biochemical and molecular mechanisms of myelination also contribute to a particular interest in studying Schwann cells. So far, the implication of LXRs and oxysterols in Schwann cell physiology has been studied using LXR null mice (LXα/β^−/−^ hereafter referred to as LXR double KO or LXRdKO) [20,21,22].

The first study exploring oxysterol/LXR signaling in Schwann cells revealed that 3 oxysterols well known to be LXR ligands (namely 24(S)-, 25-, and 27-HC) and their corresponding biosynthetic enzymes are present in adult mouse sciatic nerves as well as in a mouse Schwann cell line (MSC80) [20]. Treatment of MSC80 cells with a high concentration of 25-HC or the synthetic LXR ligand T0901317 drastically reduces the expression of two major peripheral myelin genes, myelin protein zero (*Mpz*) and peripheral myelin protein 22 (*Pmp22*), observed through a promoter transactivation assay using constructs of both the myelin genes. Interestingly, in silico analysis revealed putative LXR response element (LXRE) sites upstream of these genes, and ChIP analyses confirmed that the stimulation of LXRs increased its occupancy at these sites. Moreover, LXR activation also downregulates key components of the Wnt/β Catenin pathway, an important driver of myelination and remyelination of peripheral nerves [23,24].

However, the observations in the sciatic nerve of LXRdKO animals were interestingly different. Firstly, the transcripts of *Mpz* and *Pmp22* were upregulated complementing the results observed in MSC80. However, their protein levels were significantly lower. Moreover, the animals displayed compact but thinner myelin sheaths, possibly due to a reduction of the myelin proteins that help in maintaining the structural integrity of the sheath [25]. This issue opened up the possibility of a secondary indirect mechanism in vivo that resulted in the reduced protein expression of these myelin genes. Upon further inspection, it was found that the systemic ablation of LXRs results in a highly oxidative environment in the sciatic nerve [22]. Indeed, despite a heightened response to oxidative stress through the Nrf2 pathway, anion superoxide production, protein carbonylation, and lipid peroxidation were elevated in the sciatic nerves of LXRdKO animals. Interestingly, younger LXRdKO mice at postnatal day 21 (P21) were not affected, suggesting that the effects of the oxidative insults set in after developmental myelination is almost complete. It was thus hypothesized that in the absence of LXRs, this oxidative stress hampered the processing and the turnover of myelin proteins, especially PMP22 which is known to form dimers due to oxidative damage [26]. This phenomenon possibly leads to progressive hypomyelination during adulthood. The oxidative insults in vivo were abrogated when the mice were treated with N-ActeylCysteine (a potent reactive oxygen species scavenger) between the ages of P21 and 8 weeks. The treated mice exhibited restored protein levels of MPZ and PMP22 coupled with improvements in the thickness of the myelin sheaths and in electrophysiology. Furthermore, LXR stimulation in MSC80 Schwann cells using T0901317 was shown to ramp up the antioxidant response, thereby aiding them in surviving impending oxidative stress induced by a high dose of Tert-butyl Hydroperoxide. These results taken together suggest that LXRs negatively regulate myelin gene expression in peripheral nerves by opposing the driving effect of the Wnt/ -Catenin pathway. They also aid in maintaining the redox homeostasis in this tissue by stimulating antioxidant cellular responses.

A very recent study, using two different knockout mice models, PMP22^−/−^ and ATP Binding Cassette Transporter A1 (ABCA1)^−/−^, demonstrates a direct link between PMP22 and LXR target genes such as ATP Binding Cassette Transporter A1 (ABCA1) and Apolipoprotein E (ApoE) [27]. Increased accumulation of cholesterol at the perinuclear region of primary PMP22^−/−^ mutant Schwann cells was observed in culture along with an accumulation of lipid droplets and vesicles in mutant nerves. These observations were corroborated with a downregulation of certain genes implicated in the Sterol Regulatory Element Binding Protein (SREBP) pathway, notably Fatty Acid Synthase (FASN), HMG CoA reductase (HMGCR), and Low-Density Lipoprotein Receptor (LDLR), possibly because of a cellular response to halt lipogenesis and cholesterol synthesis following their accumulation. Targets of LXR such as ABCA1 and ApoE are possibly upregulated due to the accumulation of cholesterol as these genes are directly responsible for cholesterol efflux from the cell. However, the authors state that they do not see any modulation of LXRs in this phenomenon. The downregulation of the SREBP1c pathway, together with an upregulation of cholesterol efflux genes without any changes to LXR levels, presents a paradoxical situation as both these pathways are positively regulated by LXRs. The ABCA1^−/−^ model, on the other hand, has a converse effect with respect to PMP22. While the accumulation of lipid droplets in these mutant nerves comes as no surprise, the authors also show an increase in PMP22 protein levels coupled with improper processing of de novo PMP22 synthesis. Intriguingly, this is in stark contrast to what is observed in an LXR deficient system where the protein levels of PMP22 are downregulated [20,22]. Furthermore, both in primary Schwann cells and nerves, the authors demonstrate that ABCA1 and PMP22 proteins colocalize and can interact either directly or indirectly in a cholesterol dependent manner, without necessarily implicating LXRs.

**Figure 2:**
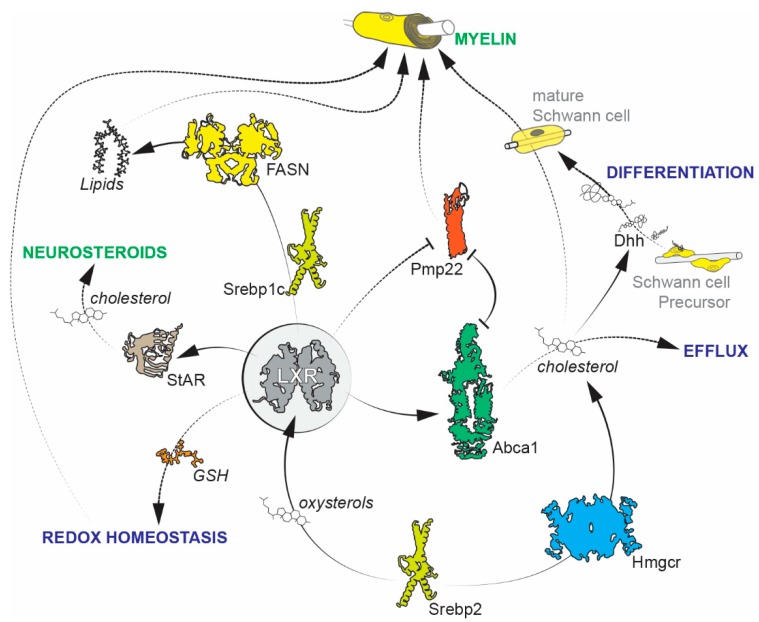
Implication of Liver X Receptors (LXRs) in several cellular pathways of Schwann cell physiology and pathology. LXRs interact with various molecular pathways that directly or indirectly impact (re)myelination. They are direct drivers of myelin gene expression (PMP22) and modulate redox homeostasis through intracellular Glutathione (GSH) levels to maintain myelin integrity. LXRs are also linked to lipid and cholesterol metabolism through the SREBP pathway. They directly regulate lipogenesis through Srebp1c and are in turn regulated by Srebp2 that is known to produce oxysterol LXR ligands to promote cholesterol efflux. Moreover, LXRs regulate neurosteroidogenesis through StAR to protect the nerve against diabetic insults. It is also possible that LXRs participate in Schwann cell development by modulating cholesterol levels in Schwann cell precursors during the processing and secretion of Dhh (refer to the “Perineurial cells” section).

In a pathological context, LXR activation has proven to be beneficial as a therapeutic approach to alleviate structural and neurophysiological anomalies of Diabetic Peripheral Neuropathy (DPN) using two distinct pathways [28,29]. In a model of streptozotocin (STZ) induced diabetes, Cermenati and colleagues first showed that STZ treatment changes the lipid composition of the myelin sheath, with notable differences in phospholipids, fatty acids, and myelin cholesterol [29]. These differences were reversed to control levels upon the treatment of STZ treated rats with a synthetic LXR agonist, GW3965. The authors further describe that the SREBP pathway, which controls lipid biosynthesis, is downregulated in STZ treated animals. SREBP1c (encoded by *Srebf1*) is a transcription factor that is located in the Endoplasmic Reticulum (ER) and shuttles to the nucleus upon activation (referred to as the active form). In STZ treated animals, ER retention was higher, and, upon LXR activation, there was an increase in nuclear translocation, thus resulting in the transcription of lipogenic genes and enzymes. They also show evidence of a restoration in MPZ protein levels, which was previously downregulated due to STZ treatment. This rescue at the molecular level also improves the myelin ultrastructure in the nerve. In another study, the authors also provide evidence of an increase in neurosteroid synthesis upon LXR activation [28]. Neurosteroids have long been known to exert a protective effect and alleviate insults that result from peripheral neuropathy [30,31,32]. In their study, Cermenati and colleagues showed that STZ induced diabetes is accompanied by a reduction in neurosteroid levels in the nerves that are concomitant with a reduction in the transcript levels of key proteins that regulate steroidogenesis from cholesterol. Notably, they show that the Steroidogenic Acute Regulatory protein (StAR), which transports cholesterol to the mitochondria for steroidogenesis, is directly controlled by LXRs. Additionally, LXR activation increases transcript levels of cytochrome P450 side-chain cleavage (P450scc) and 5α-reductase, which regulate neurosteroid synthesis. The overall effect of LXR activation resulted in a decrease in diabetic neuropathic insults at the molecular, structural, and functional levels through upregulation of protective neurosteroidogenesis. Figure 2 recapitulates the different physiological and pathological pathways implicating LXRs in Schwann cells.

### Future Avenues of Research on Schwann Cells

The physiological understanding of the role of LXRs in Schwann cell biology stems for animal models where both the isoforms of LXR are deleted systemically. Although studies point to the possible role of Schwann cells in the resulting phenotype, one cannot negate the impact and the contribution of other systems on the observed phenotype. A thorough understanding of the role of LXRs can be achieved using a Schwann cell-specific knock out. To date, only a few mutant models have targeted cholesterol and fatty acid metabolism in Schwann cells [33,34,35]. These studies have targeted crucial genes such as Sterol regulatory element-binding protein cleavage (SCAP), Fatty Acid Synthase (FASN), and Squalene Synthase (SQS) in Schwann cells, and all these mouse models develop neuropathy. Nevertheless, these targeted mutations are downstream of the LXR pathway. Although it provides us with a glimpse of what we can expect in an LXR deficient Schwann cell, a Schwann cell-specific knockout of LXRs can possibly result in a similar phenotype or a much severe one given the myriad number of interactions that LXR exhibits with other important metabolic pathways described in previous studies.

Moreover, Schwann cell pathologies open up multiple avenues of research using LXRs. The intricate association between PMP22, LXRs, lipids, and cholesterol explained herein offer unexplored possibilities of using the LXR pathway therapeutically in the treatment of Charcot-Marie-Tooth 1A (CMT1A). CMT1A in humans is characterized by a duplication of Chromosome 17p12 (c17p12), which is a large segment of DNA that encodes the PMP22 protein [36,37]. This results in higher expression levels of PMP22 and compromises the structural and functional integrity of the myelin sheath around peripheral axons. Recently, Fledrich and colleagues have shown that in rodent models of CMT1A, there is a systematic downregulation of lipogenic genes in the nerves [38]. It is interesting to note that PMP22^−/−^ animals exhibit the exact opposite phenotype with accumulation of lipids and cholesterol in Schwann cells [27]. In the CMT1A model, administration of phospholipids improved myelin ultrastructure, electrophysiology, and muscle strength of affected animals. As discussed previously, LXR activation using GW3965 resulted in an overall increase in the expression of lipogenic enzymes altered during STZ induced diabetic peripheral neuropathy [29]. A similar strategy may also prove to be beneficial in CMT1A, given the similarity of the molecular phenotype observed.

Another Schwann cell pathology that is of particular interest to the glial research community is Malignant Peripheral Nerve Sheath Tumor (MPNST), a malignant form of Neurofibromatosis Type 1 (NF1) resulting from a loss of function mutation in the Neurofibromin 1 gene [39,40]. MPNST arise from benign tumors of Schwann cell origin called plexiform neurofibromas in approximately 10% of all afflicted patients [39]. MPNSTs have been characterized with an altered lipid metabolism resulting from increased fatty acid synthesis and fatty acid oxidation—so much, so that fatty acid synthase (FASN) has been suggested to be a targetable metabolic oncogene [41]. Another approach that has rendered positive results is the inhibition of the mTORC pathway [42,43]. From a reductionist perspective, these two strategies seem like two sides of the same coin as mTORC1 is classically known to activate the SREBP pathway that ultimately controls lipogenesis [44,45]. LXRs have also been known to regulate lipogenesis through SREBP1c directly [46,47,48]. To date, no data exist on the expression levels of LXRs and their lipogenic role, if any, in NF1 tumors, including MPNST. However, inhibition of the LXR pathway to inhibit lipogenesis remains to be explored as a possible therapeutic approach for combating MPNST.

## 3. Sensory Neurons in the Ganglia

The implications of LXRs in the pathophysiology of sensory neurons has recently been elucidated in two interesting studies [49,50]. In one study conducted on the dorsal root ganglia (DRG), the authors show that LXRs protect Sodium channel 10 alpha subunit (Nav1.8 also called SCN10A) expressing sensory neurons from ER stress and also from mechanical allodynia induced by diet-related obesity [50]. The authors adopt two approaches to demonstrate the same. They first show that western diet fed mice are susceptible to ER stress in the DRG because of increased expression of ER stress markers C/EBP homologous protein (CHOP), activating transcription factor 4 (ATF4), and the spliced variant of X-box binding protein-1 (sXBP1). Activation of LXRs by the administration of GW3965 to western diet fed mice significantly lowered the expression of these genes along with a concomitant increase in LXR target genes, ABCA1 and ApoE, in the DRG. The activation also proved to be helpful in delaying the onset of induced allodynia. They also observed that saturated fatty acids, such as Palmitate, have the potential to induce ER stress in ex vivo DRG cultures. LXR activation proves to be equally helpful in reducing ER stress in ex vivo cultures. Secondly, and more importantly, the authors adopt cell-specific approaches to show that the absence of LXRα and LXRβ in Nav1.8 expressing sensory neurons increases allodynia and thermal sensitivity in these mutants and results in ER stress in DRG both in vivo and ex vivo. These results suggest that the phenotype is indeed driven by LXRs, specifically in Nav1.8 expressing sensory neurons in the DRG.

The second study was conducted in the nodose ganglia of the vagus nerve using the same cell-specific Nav1.8 Recombinase driver to ablate both LXRs. Nav1.8 sensory neurons are also situated in the nodose ganglia [50]. This study is very intriguing because it shows that a cell-specific ablation results in a whole-body phenotype. The authors first confirmed that the mutation has the desired downregulatory effect on LXR target genes *Abca1* and *Srebp1c* in the nodose ganglia. Secondly, when these mutant mice were fed a western diet, they accumulated more cholesterol in the ganglia. However, they did not seem to gain more weight after western diet when compared to controls. This was coupled with lesser body fat accumulation and no difference in lean mass. Upon further inspection, it was revealed that mitochondrial metabolism was upregulated in the brown adipose tissue due to an upregulation of genes such as Uncoupling protein 1 (*Ucp1*) and peroxisome proliferator-activated receptor gamma coactivator 1-alpha (*Pgc1*α) both at the transcript and protein levels. Additionally, mitochondrial oxidative respiration was also activated in the skeletal muscle. Thus, the authors postulated that the two observations correlate with the observed lack of weight gain in these mutants. Finally, the authors also observed a downregulation in γ synuclein (Syng) in the nodose ganglia, a protein that is known to regulate synaptic trafficking and the formation of lipid droplets. They remarked this down-regulation, and the observed phenotype concurs with the total Syng^−/−^ mouse model and that the obesity-resistant phenotype is also observed in the LXRdKO mice. The study concluded that regulation of cholesterol and lipid metabolism can have post-synaptic effects in other tissues through yet undiscovered mechanisms.

### Future Avenues of Research on Sensory Neurons

Both these studies have been conducted in mice with cell-specific ablation of both LXRα and LXRβ. However, the first study does state that the expression levels of both these isoforms are not equal in the ganglia [49]. LXRβ expression is classified as “high” and that of LXRα is classified as “moderate”, and the relative difference is not mentioned. Nevertheless, it would be interesting to study which of these isoforms is implicated more in the phenomenon observed. This line of thought is motivated by two key observations. In the CNS, LXRβ^−/−^ mice tend to show more pertinent phenotypes than LXRα^−/−^ mice, possibly because of the distribution of their expression in different regions [10]. Secondly, our personal observations in the sciatic nerves, Schwann cell-line MSC80, and primary mouse Schwann cells show that the β isoform is expressed much more the α counterpart. Therefore, it is quite possible the LXRβ is indeed more important in the physiology of both the CNS and PNS. This hypothesis, however, remains to be verified rigorously.

## 4. Endoneurial Cells

Endoneurial cells (or endoneurial fibroblast-like cells of peripheral nerves) are multifaceted glial cells which are implicated in various functions such as endoneurial structural integrity, myelin clearance after nerve injury, as well as in mediating immune responses in the peripheral nerves [51,52]. The origin of these cells was highly debated until critical observations were put forth to confirm their neural crest origin, much like Schwann cell precursors [15,53]. Although they have been well characterized with cell-specific markers in the recent years, the LXR/lipids/cholesterol axis in these cells has not been studied so far, even though these cells are indeed strong candidates.

**Figure 3:**
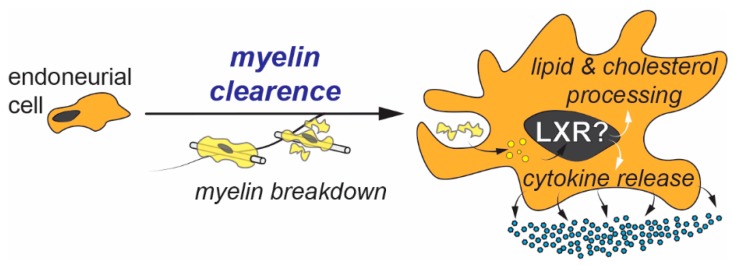
Role of Endoneurial cells in myelin clearance after injury. In response to myelin breakdown, endoneurial cells digest myelin debris through phagocytosis and release cytokines to create a proinflammatory environment. Given the capacity of LXRs to regulate lipid/cholesterol metabolism and immune responses, LXR signaling might play an important role in the physiology of these cells.

Firstly, endoneurial cells have been directly implicated in the clearance of myelin debris through phagocytosis [54,55,56]. The capacity to ingest and clear myelin debris suggests that these cells should have highly functional lipid and cholesterol regulatory mechanisms, especially during nerve regeneration after injury. The role of LXRs, if any, in these mechanisms is yet to be elucidated (Figure 3).

Secondly, endoneurial cells are also known to be the first immune responders during nerve injury and participate in creating a proinflammatory environment that favors myelin clearance and axonal regeneration [51]. Consequently, they are sometimes referred to as the “resident macrophages of the peripheral nerve” [57]. Hence, it is quite possible that immune responses mediated by these cells are also regulated by LXRs, given the latter’s implications in inflammation and immunity [8,58].

## 5. Perineurial Cells

Perineurial cells are cells that align the perineurium of the spinal nerves. They were previously thought to have mesenchymal origins and, hence, were called perineurial fibroblasts [59]. However, recent work from Kucenas and colleagues have reclassified them as perineurial cells originating at the Motor Exit Points (MEP) of the spinal cord [60]. Indeed, MEP glial cells are now studied as a separate family of PNS glial cells originating in the CNS [61,62]. These cells were previously shown to be the target cells of the Desert Hedgehog (Dhh) ligand secreted by Schwann cells during peripheral nerve development [63,64]. Indeed, improper Dhh signaling results in the invasion of the endoneurial space by perineurial cells forming mini-fascicles around groups of myelinated axons.

**Figure 4:**
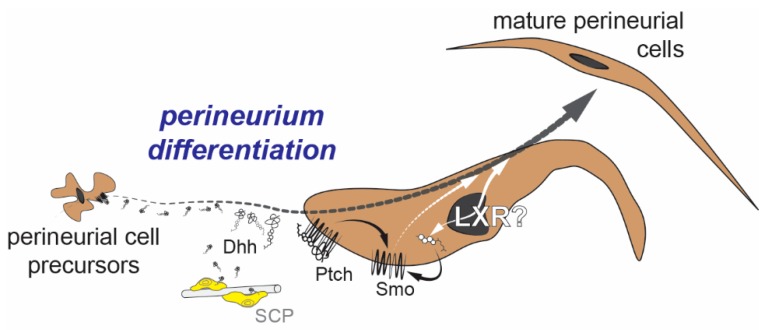
Development of perineurial cells and Desert Hedgehog (Dhh) signaling. Dhh, secreted by Schwann Cell Precursors (SCP), interacts with the Patched (PTCH) receptor at the surface of the perineurial cell precursors. It allows Smoothened (SMO) to be activated to trigger a differentiation program. To be functional, SMO also requires the presence of endogenous cholesterols and oxysterol molecules that are also potent LXR ligands. Therefore, a functional dialogue between these two signaling pathways can be speculated.

Interestingly, hedgehog signaling is intrinsically linked to cholesterol, both in the cells that secrete the protein as well as the ones receiving it [65]. In the secreting cell (Schwann cell), the Dhh protein is first cleaved, and a cholesterol moiety is attached to its C terminal. This modification helps in the transport of the protein from the cytosol to the plasma membrane where it is retained before being shunted out of the cell. In the target cell (Perineurial Cells for example), Dhh binds to its receptor Patched1 (PTCH) and releases that latter’s inhibition of Smoothened (SMO), thus activating the downstream signaling cascade of *Dhh* in target cells. The activation of SMO, however, requires the presence of endogenous cholesterol or oxysterols, some of which are potent ligands of LXR [66,67]. Therefore, it is quite probable that perineurial cells also regulate endogenous cholesterol and oxysterols through LXRs and their target genes (Figure 4). Thus, two fundamental questions remain to be answered. Do LXRs modulate cholesterol/oxysterols levels to be permissive for Dhh signaling both from the secreting cell and the target cell? Does Dhh signaling in the nerve occur simultaneously with LXR stimulation to mediate the interaction between Schwann cells and Perineurial cells during development?

## 6. Discussion

The review so far has considered the implications of LXRs in the physiology and pathology of the Peripheral Nervous System along with associated research questions that are yet to be answered. Cholesterol and lipid metabolism are fundamental biochemical processes that are essentially involved in most cell types of the body. Indeed, the different cells of the PNS utilize these metabolic processes, possibly by the same means, although for different ends. LXRs find themselves at the nexus of these two metabolic processes and, hence, understanding their physiological role in its entirety is crucial for formulating both cellular and generic therapeutic approaches. For instance, stimulating LXRs may render the desired effect of cholesterol efflux through ABCA1 and ApoE, but would also result in increasing lipogenesis through the SREBP pathway if the system permits both. This is one of the main reasons why administering LXR agonists as such as T0901317 or GW3965 for therapy might have the desired effects on the system being studied, but also promote dyslipidemia in the liver and reverse cholesterol transport systemically. Similarly, antagonizing or inhibiting LXRs could result in cholesterol accumulation and would concomitantly decrease lipogenesis in any system.

Certain plausible solutions can be envisaged to tackle this conundrum and to make LXR based therapies more tenable. Fundamental research on identifying which isoform of LXR is necessary and sufficient for a physiological function provides a better framework for designing therapies using isoform-specific agonists or antagonists. For example, in the CNS, LXRβ is known to regulate Oligodendrocyte Precursor Cell (OPC) differentiation, prevent adult motor neuron degeneration, and promote ventral midbrain neurogenesis [68,69,70]. However, LXRα is important for maintaining the blood-brain barrier [71]. Incidentally, it has also been shown that certain LXRβ agonists can have the desired effect on cholesterol metabolism without having unacceptable lipogenic effects on the liver [72]. This is mostly because LXRα, but not LXRβ, is indispensable for fatty acid and lipogenesis in the liver [73,74]. Therefore, agonizing LXRβ as a therapeutic approach is more promising in the context of CNS disorders. In the PNS, the role of the different LXR isoforms is poorly understood in different cells. However, it is quite probable that LXRβ is the functionally predominant isoform in the PNS, especially in the context of myelination, given its expression pattern.

Another solution to make successful LXR based therapies is to identify function/cell-specific ligands without any prior knowledge of their specificity to different isoforms. This has been well elucidated in the case of macrophages where desmosterol has been shown to activate cholesterol efflux through LXR in these cells, but not induce lipogenesis in the hepatocytes [75,76]. Identification of such cell or tissue-specific agonists or antagonists in the PNS would be a breakthrough in combatting peripheral nerve disorders and pathologies.

With regard to human pathologies, stimulating LXRs seems to be a possible therapeutic approach for CMT1A. The objective of such an approach would be to counteract the phenotypic decrease in lipogenesis by stimulating the LXR/SREBP1c axis. For NF1 related MPNSTs, the potentiality of LXR based therapies can only be evaluated if the lipogenic role of the nuclear receptor is completely understood both in benign plexiform neurofibromas and MPNSTs. Nevertheless, as increased lipogenesis is a hallmark of MPNST, inhibiting LXRs can be envisaged as a plausible therapeutic approach.

In conclusion, further research in the PNS should be directed towards understanding the role of LXRs at a cellular level in different glial cells of the PNS. Moreover, the identification of the functional isoform in each cell type would provide additional information in our understanding of PNS physiology. Fundamental research is also required in identifying local, tissue-specific natural agonists or antagonists of LXR. These advances would permit us to assess the true potential and promise of LXR based therapies for different pathologies of the PNS.
